# Distance-Based Configurational Entropy of Proteins from Molecular Dynamics Simulations

**DOI:** 10.1371/journal.pone.0132356

**Published:** 2015-07-15

**Authors:** Federico Fogolari, Alessandra Corazza, Sara Fortuna, Miguel Angel Soler, Bryan VanSchouwen, Giorgia Brancolini, Stefano Corni, Giuseppe Melacini, Gennaro Esposito

**Affiliations:** 1 Dipartimento di Scienze Mediche e Biologiche, Universita’ di Udine, Piazzale Kolbe 4, 33100 Udine, Italy; 2 Istituto Nazionale Biostrutture e Biosistemi, Viale medaglie d’Oro 305, 00136 Roma, Italy; 3 Department of Chemistry and Chemical Biology, McMaster University, 1280 Main St. W. Hamilton, ON L8S 4M1, Canada; 4 Center S3, CNR Institute Nanoscience, Via Campi 213/A, 41125 Modena, Italy; Consiglio Nazionale delle Ricerche, ITALY

## Abstract

Estimation of configurational entropy from molecular dynamics trajectories is a difficult task which is often performed using quasi-harmonic or histogram analysis. An entirely different approach, proposed recently, estimates local density distribution around each conformational sample by measuring the distance from its nearest neighbors. In this work we show this theoretically well grounded the method can be easily applied to estimate the entropy from conformational sampling. We consider a set of systems that are representative of important biomolecular processes.

In particular:

- reference entropies for amino acids in unfolded proteins are obtained from a database of residues not participating in secondary structure elements;
- the conformational entropy of folding of *β*2-microglobulin is computed from molecular dynamics simulations using reference entropies for the unfolded state;
- backbone conformational entropy is computed from molecular dynamics simulations of four different states of the EPAC protein and compared with order parameters (often used as a measure of entropy);
- the conformational and rototranslational entropy of binding is computed from simulations of 20 tripeptides bound to the peptide binding protein OppA and of *β*2-microglobulin bound to a citrate coated gold surface.

This work shows the potential of the method in the most representative biological processes involving proteins, and provides a valuable alternative, principally in the shown cases, where other approaches are problematic.

## Introduction

Entropy, weighted by the system temperature and counterbalanced by enthalpy is key in predicting the outcome of natural processes through the well-known Gibbs equation. At the microscopic level it determines the behavior of cell’s complex machinery by regulating proteins’ biophysical processes, such as folding and binding. These depend on several thermodynamic contributions to the free energy so that when the stabilization of structures causes large decreases of the entropic contribution, its understanding becomes essential to streamlining and controlling such processes.

Notwithstanding the efforts in this field the experimentally available information about the enthalpy and entropy of processes involving proteins or peptides is still not completely understood because it arises from several different contributions (electrostatic, hydrophobic, vibrational and from more extended molecular flexibility) which are difficult to disentangle.

In the following the configurational entropy will be addressed and the entropy arising from the different conformations of a molecule will be referred to as “conformational entropy” as usual in protein thermodynamics literature.

NMR has given a first idea of how relevant can conformational entropy be in protein ligand association by linking entropy changes to changes in measured order parameters (from protein relaxation data) or more generally protein dynamics [1, 2], but the quantification suffers from modeling and from the atomic probes available which are typically backbone HN internuclear vectors. More recently an NMR experiment able to provide entropy variations corresponding to global or partial unfolding has been proposed [3, 4], but still, although local processes can be addressed, it is impossible to disentangle conformational from solvation entropies.

Theoretical methods have been recently reviewed [5–7] in the context of protein-ligand complex formation and an in-depth discussion of the thermodynamics of binding, including a discussion of all entropic contributions, has been given by Gilson, McCammon and coworkers [8, 9].

In this respect molecular dynamics simulations have been seen as a way to compute conformational entropic contributions from the analysis of sufficiently long trajectories. Unfortunately the comparison with experimental data in this field is not straightforward because as the temperature is varied (as done in experiments aiming at measuring entropy) hydrophobic and electrostatic solvation energies are significantly changed. Also, at higher temperature protein structure becomes more loose and more conformational space may become available beyond what expected e.g. for an ensemble of oscillators.

Methodologies developed or at least fully exploited more or less recently could change the situation and make it possible to assess the various contribution to the folding or binding entropy from molecular dynamics simulations. In particular:

1. solvation terms could be obtained by the accurate implicit solvent representation based on the Onufriev Bashford and Case approximation to volume integrals [10], which allows also to speed up significantly simulations;
2. advanced sampling methods, reviewed by Adcock and McCammon [11] which allow on one hand a better sampling of the conformational space of the simulated systems and on the other hand, with some methodologies, to assess the effect of temperature on the conformational space of the molecule under study [12];
3. and lastly, the *k* ^*th*^ nearest-neighbor estimate of entropy for a general probability distribution, proposed by Demchuk and collaborators [13] and developed by Hnizdo, Gilson and others [14–16], that allows an efficient estimation of entropy for multivariate probability distributions. The latter method has been applied mainly by the developers and few other groups [14–23].

In this work we explore the application of the nearest-neighbor method to estimate the entropy for a few systems that are representative of important biomolecular processes like folding, allosteric transitions and ligand binding. Although results cannot be directly compared with experimental data, they agree with previous theoretical estimates and in all cases are in the expected range.

The present work adds to the few works that make use of the approach of Demchuk and coworkers, showing its potential in most common applications and develops to some extent the subject by addressing the entropy of folding and the rototranslational entropy loss upon binding.

The paper is organized as follows:

- methods are first reviewed and linked to the formalism used here;
- reference entropies for amino acids in unfolded proteins are obtained from a database of residues not participating in secondary structure elements;
- the conformational entropy of folding of *β*2-microglobulin [24–26] is computed from molecular dynamics simulations after subtraction of the reference entropies for the unfolded state;
- backbone conformational entropy is computed for molecular dynamics simulations of four different states of the EPAC protein [2, 27, 28] and compared with order parameters (often used as a measure of entropy) and overall conformational entropy;
- the conformational and rototranslational entropy of binding is computed from simulations of 20 tripeptides bound to the peptide binding protein OppA [29, 30] and of *β*2-microglobulin bound to a citrate coated gold surface.
- finally the results and the comparison with experimental data are discussed.

The main conclusion of this work is that for a set of systems representative of important biomolecular processes, where other approaches to the computation of entropies are problematic, the method based on the nearest neighbor can be readily applied to molecular dynamics trajectory as a valid alternative to other widely used methods.

## Materials and Methods

### The thermodynamic potential of a solvated molecule

In this subsection we recall the basic equations for the thermodynamic potential of a solvated molecule, following the book of McQuarrie [31] and the review of Gilson et al. [8] and Roux and Simonson [32]. We will use in the following molecular instead of molar quantities.

The standard chemical potential is expressed in terms of configurational integrals:

$$\begin{array}{c} \mu_{A}^{0}=-k_{B}T\log(\frac{8\pi^{2}}{C^{0}}\prod {\left(\frac{2\pi m_{i}^{A}k_{B}T}{h^{2}}\right)}^{\frac{3}{2}}\int exp\left(-\beta \left(U\left({\vec{r}}_{A}\right)+\Delta G_{solv}\left({\vec{r}}_{A},T\right)\right)\right)d{\vec{r}}_{A})+\frac{P^{0}{\bar{V}}_{A}}{N_{Av}} \end{array}$$ (1)

with $\beta =\frac{1}{k_{B}T}$, where *k*_*B*_ is the Boltzmann constant and T is the temperature, *h* is Planck’s constant, $m_{i}^{A}$ the mass of atom *i* of the solute and solute (and solvent, in the following) coordinates are indicated by ${\vec{r}}_{A}$ (and ${\vec{r}}_{S}$, respectively). *P* ^0^ is the standard pressure, ${\bar{V}}_{A}$ the partial molar volume of the solute, *C* ^0^ is the standard 1 M concentration and *N*_*Av*_ is Avogadro’s number.

The solvation potential of mean force $\Delta G_{solv}({\vec{r}}_{A},T)$ is defined by the configuration integral:

$$\begin{array}{c} exp\left(-\beta \Delta G_{solv}\left({\vec{r}}_{A},T\right)\right))=\frac{\int exp(-\beta \left(U_{AS}\left({\vec{r}}_{A},{\vec{r}}_{S}\right)+U_{S}\left({\vec{r}}_{S}\right)\right)d{\vec{r}}_{S}}{\int exp\left(-\beta U_{S}\left({\vec{r}}_{S}\right)\right)d{\vec{r}}_{S}} \end{array}$$ (2)

In what follows we will not consider the kinetic energy terms in the partition functions because they will cancel in processes involving isolated proteins, and the term $P^{0}{\bar{V}}_{A}$ will be neglected because it is typically small for proteins and its variations upon conformational changes are negligible. For instance upon folding protein volume changes by less than 0.5 percent at standard pressure [33], which amounts to a mean free energy variation of just about 20 J/mol for a protein of 50 kDa. The picture would obviously change completely at high pressures.

We may use the probability distribution

$$\begin{array}{c} p\left({\vec{r}}_{A},T\right)=\frac{exp(-\beta \left(U\left({\vec{r}}_{A}\right)+\Delta G_{solv}\left({\vec{r}}_{A},T\right)\right))}{\left(\int exp(-\beta \left(U\left({\vec{r}}_{A}\right)+\Delta G_{solv}\left({\vec{r}}_{A},T\right)\right))d{\vec{r}}_{A}\right)} \end{array}$$ (3)

to express the entropy as:

$$\begin{array}{c} S=-k_{B}\int p\left({\vec{r}}_{A},T\right)\log\left(p\left({\vec{r}}_{A},T\right)\right)d{\vec{r}}_{A}-\int \frac{\partial \Delta G^{solv}\left({\vec{r}}_{A},T\right)}{\partial T}p\left({\vec{r}}_{A},T\right)d{\vec{r}}_{A} \end{array}$$ (4)

The first term is a classical conformational entropy term for the solute.

The second term is the entropy due to the solute-solvent interactions.

Note that the latter term cannot be simply estimated from the known or modeled dependence of $\Delta G^{solv}({\vec{r}}_{A},T)$ on the temperature because it depends also on the conformational space available which depends in turn on the temperature.

In the present work we are interested in the characterization of the first term, i.e. the entropy term arising from the conformational freedom of the protein.

### Calculation of entropy from samples in conformational space

We follow here the approach of Singh et al. [13] developed and applied by Hnizdo, Gilson and coworkers [14, 15, 34, 35]. The approach considers *n* configurational samples for a random *s*-dimensional variable distributed according to the probability density *p*(*x*_1_, *x*_2_, …, *x*_*s*_) (or $p(\vec{x}))$ for short). Given an *s*-dimensional sphere of radius *r* centered at $\vec{x}$ a reasonable estimate for the local probability density ($\hat{p}(\vec{x})$) may be expressed by counting how many samples are found inside the volume of the sphere *V*_*r*_. If this number is *k*, then $\hat{p}(\vec{x})$ is reasonably defined by the equation:

$$\begin{array}{c} \hat{p}\left(\vec{x}\right)V_{r}=\frac{k}{n} \end{array}$$ (5)

Note that in general *V*_*r*_ depends on the metric defined on the space which is considered. For euclidean distances the volume is given by:

$$\begin{array}{c} {\hat{V}}_{r}=\frac{\pi^{\frac{s}{2}}r^{s}}{\Gamma (\frac{s}{2}+1)} \end{array}$$ (6)

where Γ() is the Γ function.

With an estimate for the density the entropy can be estimated (we omit here the *k*_*B*_ factor for generality) as the average of $\text{log}\;p(\vec{x})$:

$$\begin{array}{c} S=-\int p\left(\vec{x}\right)\log p\left(\vec{x}\right)\approx -\frac{1}{n}\sum_{i}\log(\hat{p}\left({\vec{x}}_{i}\right)) \end{array}$$ (7)

For each sample *i* we take a sphere of radius *r*_*i*_ and count the number of samples *k*_*i*_ found in the sphere. By substituting Eqs 5 and 6 into the above equation we can reasonably approximate entropy by:

$$\begin{array}{c} S\approx \frac{1}{n}\sum_{i}\log(\frac{n\pi^{\frac{s}{2}}r_{i}^{s}}{k_{i}\Gamma \left(\frac{s}{2}+1\right)})=\frac{s}{n}\sum_{i}\log\left(r_{i}\right)+\log\left(\frac{n\pi^{\frac{s}{2}}}{\Gamma (\frac{s}{2}+1)}\right)-\frac{1}{n}\sum_{i}\log\left(k_{i}\right) \end{array}$$ (8)

In the approach described by Singh et al. [13] the radius of the sphere centered about each conformational sample is taken as the distance to the *k*-th nearest neighbor and *k*_*i*_ is therefore the same for all samples. The above heuristic equations are corrected as to provide an unbiased estimator of the entropy for the probability distribution $p(\vec{x})$:

$$\begin{array}{c} {\hat{S}}_{k}^{\left(n\right)}=\frac{s}{n}\sum_{i=1,n}\log R_{i,k}+\log\frac{n\pi^{\frac{s}{2}}}{\Gamma (\frac{1}{2}s+1)}-L_{k-1}+\gamma \end{array}$$ (9)

In the above equation *R*_*i*,*k*_ is the distance of the *i*-th sample from its *k*-th nearest neighbor, Γ() is, as before, the Γ function, *γ* is Euler’s constant (0.5772…) and *L*_*k*−1_ is defined as: *L*_0_ = 0, $L_{j}=\sum_{i=1,j}\frac{1}{i}$.

It is easy to recognize the similarity of the heuristic and exact equation.

Note that *R*_*i*,*k*_ has a unit of measure and therefore the numerical value of ${\hat{S}}^{\left(n\right)}$ depends on the latter unit. This is consistent with the fact that in the equation for the entropy *S* = −*k*_*B*_∫*p*log(*p*) the probability density *p* has units of measure.

To illustrate the symbols in the above equation consider a long molecular dynamics trajectory for a small molecule possessing only two rotatable bonds. The *i*-th (out of 1000) sample of the conformational space could be represented by the 2 torsional angles describing its conformation (assuming rigid bonds and angles) taken from the *i*-th snapshot. In this case the dimensionality of the system *s* would be 2 and the number of samples *n* would be 1000.

The distance *d* of each sample from all other samples would be computed according to the metric chosen (for torsional angles this is described below), and ranked, e.g. for the *i*-th snapshot *R*_*i*,*k*_ would be then the *k*-th shortest computed distance. For each choice *k* we could use Eq (9) to estimate the configurational entropy of the molecule. In the following, unless otherwise stated, we will use *k* = 1, i.e. for each sample we consider the distance from its first nearest neighbor.

### Limitations in the sampling capabilities of molecular dynamics simulations

A caution word is due about the possibility of sampling the conformational space of a molecule by molecular dynamics simulations. Indeed typical simulation times extend up to a hundred nanoseconds. It is likely that on this time scale sampling of the conformational space will not be complete. This consideration will apply to the approach used in this work as well as to other approaches found in the literature.

When comparing the entropy of different macrostates of the same molecule the effect of neglected accessible conformational space could be greatly reduced if the missing portion of sampled conformations is similar in the two simulations.

We will not address the issue here in detail as it affects similarly all methods that estimate conformational entropy from molecular dynamics simulations snapshots. For the shortest simulations we will only check convergence by considering the first and the last half of the simulations and compare the results obtained from the two subsets of snapshots. Even such test does not guarantee that other conformations will not be accessible in longer simulation times.

The main interest here however is a proof of principle and to show that even from standard simulations it is possible to get valuable information about conformational entropy.

### Calculation of the conformational entropy from molecular dynamics simulations

A widespread approach to compute the conformational entropy of a protein uses semi-classical quasi-harmonic analysis [36–41] or, in a refined approach, fitting probability distributions (see e.g. [42, 43]). Here we assume that the largest changes in the conformational entropy of a protein in most interesting biological processes arise from energetic restraints in the torsional freedom of backbone and sidechain groups. For this reason we consider torsional angles as variables and we neglect bond lengths and bond angles. The latter approximation amounts to assuming that the torsional degrees of freedom are decoupled from bond and angle degrees of freedom, which is mostly a good approximation for proteins, and that the vibrational partition function of bonds and angles is not significantly changed by conformational changes [38].

The latter observation might be system dependent. To give an estimate of the quality of this approximation, we considered the particular case of the residue undergoing the largest changes in conformational freedom between the holo/active and apo/inactive EPAC simulations described later. We computed the entropy using the method of Schlitter [40] as implemented in the software Carma [44] from molecular dynamics trajectories fixing or not the bond lengths and angles to their equilibrium values. In this way we were able to assess the bond and angle relative contributions to the entropy. The change in entropy between holo/active and apo/inactive, after fitting the residue to the first snapshot, was 0.264 kJ/(mol K) for free covalent geometry and 0.279 kJ/(mol K) for fixed covalent geometry. The effect appears therefore rather limited, compared to the overall computed entropy or the effect of the fitting reference chosen. This is in line with the observed changes in bond length and angle variances (about 6% with different signs). Note that the computed entropy change appears artifactually very large, possibly as a consequence of the adopted harmonic oscillator model.

We consider only the torsional degrees of freedom involving heavy atoms or polar hydrogens of serine, threonine, tyrosine, whereas proline, aromatic rings, amide and guanidinium moieties are taken as rigid. Other terminal hydrogens that could define torsional angles, but possess ternary symmetry, i.e. methyl and amino groups are considered free to rotate (or more generally we assume that their conformational distributions are not affected by the processes considered here) and therefore the contribution to the entropy of any conformational change will be zero for these moieties. For torsional angles distances we use the distance *d*_1_ (*d* for simplicity here) studied in the work of Hnizdo and coworkers [20], which amounts to the circular distance in the torsional angle space. For two angular vectors ${\vec{\theta }}_{1}=(\theta_{11},\theta_{12},...\theta_{1n})$ and ${\vec{\theta }}_{2}=(\theta_{21},\theta_{22},...\theta_{2n})$ of *n* angles each, and with periodicities Θ_*i*_ the distance is defined as:

$$\begin{array}{c} d=\sqrt{\sum_{i=1,n}{\left(\frac{\Theta_{i}}{2}-|\frac{\Theta_{i}}{2}-|\theta_{1i}-\theta_{2i}||\right)}^{2}} \end{array}$$ (10)

with *θ*_1*i*_ and *θ*_2*i*_ reported in the interval $\left[-\frac{\Theta_{i}}{2},\frac{\Theta_{i}}{2}\right]$ It must be noted that on changing from cartesian to BAT (bond, angle, torsion) coordinates the Jacobian of the transformation enters the configurational integral [8, 45]. Under the assumption of rigid bond and angle geometry this extra term cancels out between reference (or initial) and final state of the system. The proof of independence of the Jacobian from the configuration has been given by Go and Scheraga [46] cited in ref. [45] for a linear chain. The proof can be extended to a branched chain and for an arbitray atom taken as the reference point for global translation. For this reason it will not be further considered here.

### Nearest neighbor estimate of entropy is superior to classical harmonic analysis

It might be questioned whether the approach used here is needed at all when other methods are widely used. Most widely used methods are based on the quasi-harmonic approximation. The limitations and remedies to the quasi-harmonic approximation have been thoroughly described by McCammon and coworkers [42, 43] and Numata et al. [18]. We will not deal with this subject here but just provide a simple example demonstrating the issue.

If a system freely moving in one-dimension is considered and the range of movement is restricted, the quasi-harmonic approximation will give the correct change of entropy upon restriction. Indeed the square root of the variance for a uniform distribution is proportional to the range of the distribution itself. However it is sufficient to consider a toy model where we have a single freely rotatable bond (torsion angle *θ*) with three atoms fixed and the end atom moving, to see that the quasi-harmonic approximation breaks down.

We consider the unrestricted rotation as the reference state (probability density $\frac{1}{2\pi }$ and a restricted rotational macrostate where the rotational angle is freely varying between *θ*_1_ and *θ*_2_ with Δ*θ* = *θ*_2_ − *θ*_1_. The entropies of the two probability distributions are:

$$\begin{array}{c} S_{f}=-k_{B}\int p_{f}\log\left(p_{f}\right)=k_{B}\log\left(2\pi \right) \end{array}$$

and

$$\begin{array}{c} S_{r}=-k_{B}\int p_{r}\log\left(p_{r}\right)=k_{B}\log\left(\Delta \theta \right) \end{array}$$

with the difference in entropy from the free to the restricted macrostate being

$$\begin{array}{c} \Delta S=-k_{B}\log\left(\frac{2\pi }{\Delta \theta }\right) \end{array}$$

When we compute (classical) entropies using the harmonic approximation we must compute the variance-covariance matrix for two coordinates. The analysis will result in two harmonic oscillators whose classical entropy change from free to restricted macrostate is overestimating the true entropy for all choices of Δ*θ*. In particular for a reasonable restriction to an interval of Δ*θ* = *π*/3 the entropy is overestimated roughly by a factor 2, whereas it is estimated with an average root mean square error of 5 percent by the nearest neighbor method with just one hundred samples.

### Calculation of unfolded proteins conformational entropy from database structures

Crystallographic structures of proteins are generally the most representative conformations adopted by proteins in their range of stability, and for this reason are considered a good representation of the native protein ensemble. However, the conformational preferences of amino acids observed experimentally in the folded structure are not excluded from the conformational diversity of the set of partially folded and unfolded conformations that proteins can adopt. Indeed it is reasonable to assume that a reversible process like folding (at least for small proteins) simply freezes a set of amino acid conformations which can be adopted in the unfolded protein ensemble.

This principle has driven the refinement of available forcefields [47–49] and received an important validation from long time molecular dynamics simulations where the superiority of forcefield refined against experimental torsion angles distributions, and further refined afterwards, were able not only to fold small protein domains, but also to reproduce their folding rates [49, 50].

Based on the above considerations, we collected all possible conformations of all residues not directly involved in secondary structures of proteins, thus avoiding artifacts due to correlations, in the culled pdb dataset (3600 proteins) [51], as they provide samples of the conformational space available to aminoacids in their unfolded state, averaged over sequence details. This procedure assumes the presence of an identical interaction between the neighboring residues (i.e. the rest of the chain) and the unfolded and/or the database samples.

Therefore additional correlations between aminoacids in the unfolded state are not considered.

It should be noted that obtaining the same information from molecular dynamics simulations would not be easy because of the presumably largely asymmetric simulation box.

Obviously the accessible conformational space depends on the temperature, but it is reasonable to expect that this dependence is limited for stable proteins in their stability range. Indeed we can consider that for each torsional angle there is a number of preferred values (rotamers) and that the range of values explored about the most frequent ones increases with temperature. This situation is very similar to that of the harmonic oscillator. For a harmonic oscillator with frequency *ν* the vibrational partition function *q*_*vib*_, the corresponding entropy *S*_*vib*_ and its derivative with respect to temperature, in the high temperature limit are given by:

$$\begin{array}{c} q_{vib}=\frac{k_{B}T}{h\nu } \end{array}$$ (11)

$$\begin{array}{c} S_{vib}=k_{B}+k_{B}\log\left(\frac{k_{B}T}{h\nu }\right) \end{array}$$ (12)

$$\begin{array}{c} \frac{\partial S_{vib}}{\partial T}=\frac{k_{B}}{T} \end{array}$$ (13)

If we approximate the effect of temperature on the conformational landscape of unfolded proteins with that of a collection of *N* harmonic oscillators, where *N* is the number of torsional degrees of freedom we obtain:

$$\begin{array}{c} \frac{\partial S}{\partial T}\approx \frac{Nk_{B}}{T} \end{array}$$ (14)

We may therefore expect that the dependence of the computed entropy on the temperature would be rather mild. We consider a typical range of temperatures of 30 K around 300 K, where most proteins are stable. To avoid misunderstanding we remark that here we are considering the effect of temperature on the variability of each single rotamer, i.e. about a well defined torsional energy minimum, and not different rotamers which are already sampled in the dataset.

The expected change in the entropy (per oscillator) corresponding to changes in distribution about the minimum energy rotamers due to temperature are limited by $k_{B}\frac{30}{300}$ which appears negligible compared to the overall entropy associated with the loss of conformational freedom.

All database proteins have been processed adding hydrogens by the program Reduce [52]. Torsional angles have been computed using the program Molmol [53]. Only rotatable torsional angles involving four heavy atoms or three heavy atoms and a polar hydrogen have been considered.

For polar hydrogens only residues involved in hydrogen bonds have been considered in order to avoid large artifactual entropy contributions from the preference in trans conformation for the program Reduce in the absence of favorable interactions. This choice was adopted for tyrosines, serines, threonines and cysteines. Cysteines involved or not in disulfide bridges were considered separately for analysis.

Distances in the torsional angles space are computed by taking euclidean distances in the periodic boundary *n*-dimensional angular space. In order to reduce the large conformational space, torsion angles involving different aminoacids have been considered as independent and therefore entropies are computed on a residue basis. The entropy corresponding to each aminoacid type, whose conformation is defined by the *n*–dimensional vector of torsion angles *θ*, is calculated with reference to the uniform distribution in the angular space, i.e.:

$$\begin{array}{c} p_{ref}\left(\vec{\theta }\right)=\prod_{i=1,n}\frac{1}{\Theta_{i}} \end{array}$$ (15)

$$\begin{array}{c} S_{ref}=k_{B}\sum_{i=1,n}\log(\Theta_{i}) \end{array}$$ (16)

where Θ_*i*_ is the periodicity of the *i*-th torsional angle, typically 2*π*.

Note that the volume of a *n*-dimensional sphere enters Eq (9) [34] and therefore suitable corrections to the equation should be applied when considering distances larger than half of the minimum periodicity of the torsional angles. This was not the case for our analysis because of a sufficiently large number of samples, leading to short nearest neighbor distances. Exact formulae have been reported by Hnizdo and coworkers [20].

### The conformational entropy of folding

Wildtype *β*2-microglobulin was simulated for 30 ns, after equilibration at 320 K as previously described [54].

The entropy for each set of conformations was computed using Eq (9) with reference to the uniform torsion angle distribution exactly as in the previous section, taking 300 snapshots from the 30 ns molecular dynamics simulation. Then, the reference entropy computed from residues in the database representing unfolded amino acids was subtracted from the computed entropy, and the difference was taken as the conformational entropy of folding.

### The conformational entropy of binding

The calculation of the binding free energy of association of biomolecules is very complex due to the large number of interacting bodies. In order to understand and possibly design novel interactions the free energy of binding is often approximated by the sum of different contributions, under the assumption that they can be decoupled. A derivation of this approach and an exhaustive discussion of its limitations has been given by Gilson et al. [8]. Typically the attention is focused on solute degrees of freedom and solvent degrees of freedom are taken into account using the potential of mean force [32]. Evaluation of the entropy loss upon interaction is not straightfoward because the potential of mean force depends implicitly on the temperature.

We take the derivative of the Gibbs’ free energy:

$$\begin{array}{c} S_{A+B\to AB}^{0}=-\frac{\partial \Delta G_{A+B\to AB}^{0}}{\partial T} \end{array}$$ (17)

where:

$$\begin{array}{c} \Delta G_{A+B\to AB}^{0}=\mu_{AB}^{0}-\mu_{A}^{0}-\mu_{B}^{0} \end{array}$$ (18)

The choice of coordinates must allow easy calculation of the probability distribution *p*(). In this respect, following Gilson et al. [8], we choose the cartesian coordinates ${\vec{r}}_{A}$ and ${\vec{r}}_{B}$ of a reference atom on molecules A and B, respectively and other two atoms, assumed to be rigidly positioned with respect to the formers, on each molecule, to define the overall rotation state of the molecule.

We use as a criterion that the choice of the atoms must be the most insensitive with respect to dynamics in the bound state. This is done in order to minimize the correlation of these coordinates with other degrees of freedom, and therefore to decouple them from internal degrees of freedom.

For the complex we consider the coordinate system defined for molecule A as the reference one and we define the position of molecule B relative to ${\vec{r}}_{A}$, i. e. the position of molecule B is defined by ${\vec{r}}_{B}^{\prime }={\vec{r}}_{B}-{\vec{r}}_{A}$. The entropic contribution due to loss in rototranslational freedom is computed considering that all positions and orientations are still possible for the complex whereas the position and the orientation of molecule *B* is restricted, with respect to the position and orientation of the reference system of coordinates of molecule A.

Let us now denote by *ξ*_*A*_ and *ξ*_*B*_ the overall rotational degrees of freedom and by ${\vec{x}}_{A}$ and ${\vec{x}}_{B}$ the internal degrees of freedom.

With this notation the entropy of complex formation may be then written as:

$$\begin{array}{ccc} S_{A+B\to AB} & = & -k_{B}\int_{bound}p\left({\vec{r}}_{B}^{\prime },\xi_{B},T\right)\log\left(p\left({\vec{r}}_{B}^{\prime },\xi_{B},T\right)\times 8\pi^{2}V_{0}\right)d{\vec{r}}_{B}^{\prime }d\xi_{B} \end{array}$$ (19)

$$\begin{array}{ccc} & & -k_{B}\int_{bound,internal}p\left({\vec{x}}_{A},{\vec{x}}_{B},T\right)\log\left(p\left({\vec{x}}_{A},{\vec{x}}_{B}\right)\right)d{\vec{x}}_{A}d{\vec{x}}_{B} \end{array}$$ (20)

$$\begin{array}{ccc} & & +k_{B}\int_{free,internal}p\left({\vec{x}}_{A},T\right)\log\left(p\left({\vec{x}}_{A}\right)\right)d{\vec{x}}_{A} \end{array}$$ (21)

$$\begin{array}{ccc} & & +k_{B}\int_{free,internal}p\left({\vec{x}}_{B},T\right)\log\left(p\left({\vec{x}}_{B}\right)\right)d{\vec{x}}_{B} \end{array}$$ (22)

$$\begin{array}{ccc} & & -\int_{bound}\frac{\partial \Delta G_{solv}\left({\vec{x}}_{A},{\vec{x}}_{B},T\right)}{\partial T}p\left(x_{A},{\vec{x}}_{B},T\right) \end{array}$$ (23)

$$\begin{array}{ccc} & & +\int_{free}\frac{\partial \Delta G_{solv}\left({\vec{x}}_{A},T\right)}{\partial T}p\left(x_{A},T\right)d{\vec{x}}_{A} \end{array}$$ (24)

$$\begin{array}{ccc} & & +\int_{free}\frac{\partial \Delta G_{solv}\left({\vec{x}}_{B},T\right)}{\partial T}p\left(x_{B},T\right)d{\vec{x}}_{B} \end{array}$$ (25)

where the integrals over the bound state require a definition of the bound state, which is typically easy to attain if the bound state has a deep energy minimimum. Otherwise it may be chosen as to adhere to experimental signal distinguishing bound from free molecules.

The first term of the above equation is difficult to treat and it has been traditionally estimated assuming that rotational and translational degrees of freedom are independent. The latter assumption seems however poorly grounded and mostly motivated by the difficulty of treating the two degrees of freedom together. We will follow however the same approach here for simplicity.

Note that however, in order to use a distance based estimation of the entropy, a distance between rotation states must be defined. Rotation states are denoted equivalently by quaternions or 3 × 3 rotation matrices. There are several metrics which can be defined in the space of rotations [55]. It must be noted that Eq (9) assumes an euclidean distance in a cartesian space and therefore the number of points of randomly distributed n-dimensional variables within an n-dimensional distance *d* increases as $\frac{n^{\frac{p}{2}}d^{p}}{\Gamma (\frac{p}{2}+1)}$.

Once a metric is defined we should compute the volume in rotation space corresponding to each distance and substitute it in the original Eq (9).

If we consider the representation of rotations which specifies the polar and azimuthal angles *ϕ*, *ψ* of the rotation axis and the rotation angle *θ*, the probability measure for a uniform distribution in rotation space is that with the axis of rotation uniformly distributed over the solid angle 4*π* and the rotation angle *θ* distributed in the range [0, *π*] with probability density [56]:

$$\begin{array}{c} \frac{2}{\pi }\sin^{2}\left(\frac{\theta }{2}\right)=\frac{1}{\pi }\left(1-\cos\left(\theta \right)\right) \end{array}$$ (26)

The metric we have used defines the distance *d* between two rotations described by 3 × 3 matrices *R*_1_ and *R*_2_ as:

$$\begin{array}{c} d=\arccos\frac{\text{Tr}\left(R_{2}^{-1}R_{1}\right)-1}{2}=\bar{\theta } \end{array}$$ (27)

where Tr is the trace operator, $\bar{\theta }$ is the angle of rotation about the rotation axis for the composite rotation $R_{2}^{-1}R_{1}$.

With this metric the volume in rotation space is:

$$\begin{array}{c} \frac{4\pi^{2}}{\pi }\int_{0}^{\bar{\theta }}\left(1-\cos\left(\theta^{\prime }\right)\right)d\theta^{\prime }=4\pi \left(\bar{\theta }-\sin\bar{\theta }\right) \end{array}$$ (28)

Note that here we considered rotations in a range of *π* and not 2*π* to avoid double counting of rotations, because a rotation by an angle *θ* about the vector $\vec{v}$ is the same as the rotation by an angle −*θ* about the vector $-\vec{v}$.

### Comparison with other methods

Entropies were also calculated according to other methods to provide comparison. A simple histogram method was applied where the range of the variables is divided in bins and the probability distribution is approximated as a piece-wise constant function on the bins. The constant value of the approximated probability distribution ${\hat{p}}_{i}$ within bin *i* is the ratio of the number of counts *n*_*i*_ in the bin over the total number of counts *n*_*c*_, i. e. ${\hat{p}}_{i}=\frac{n_{i}}{n_{c}}$. The entropy is computed as $\hat{S}=-\sum_{i}p_{i}\text{log}p_{i}$ with reference to the uniform distribution of the same number of counts on a specified interval.

For translational entropies the set of vectors was histogrammed in 3D and the entropy computed with respect to the standard 1 M reference state. The width of the 3D bins was 0.133 Å ×0.133 Å ×0.133 Å for the results reported here.

For rotational entropies the rotation matrices were converted into a set of three angles: two (*ϕ*, *ψ*) to specify the rotation axis, and one (*θ*) to specify the rotation around that axis. *θ* was allowed to vary only between $-\frac{\pi }{2}$ and $\frac{\pi }{2}$. Since the random distribution of rotation is not uniform over the three angular ranges, the range was divided in such a way that the integral over each bin was uniform. With this prescription the three angular ranges were divided in 10 × 10 × 100 bins, respectively. The reference state was the uniform distribution of rotations.

As mentioned above, the method due to Schlitter [40] estimating entropies from the covariance matrix was used as implemented in the software Carma [44]. The source code was modified as to plot the contributions of each eigenvector to the entropy.

### Molecular dynamics simulations

#### *β*2-microglobulin

Molecular dynamics simulations of *β*2-microglobulin were performed essentially as previously described [54]. Protons were added to the starting molecular structure (PDB code: 3HLA, chain B) [25] using the program pdb2gmx in the GROMACS software package [57]. Forcefield parameters (CHARMM v.27 [58] with the CMAP correction [47]) were assigned using the psfgen utility of the NAMD simulation software [59]. Ions were added as previously described [54] and TIP3P water [60] was added using the solvate module of the program VMD [61]. The simulation box was ca. 290000 Å^3^ and the number of atoms was 29638.

The temperature was set to 320 K, well below the *β*2-microglobulin melting temperature (ca. 330 K), and controlled using Langevin Dynamics with a relaxation rate of 1 ps^−1^. The pressure was set to 1.01325 bar and controlled using the Langevin Piston method [62, 63] with an oscillation time and a decay time of 100 fs. The timestep was 1 fs for bonded interactions, 2 fs for nonbonded interactions and 4 fs for long range electrostatic interactions.

10 ns equilibration followed by 30 ns production molecular dynamics simulations were run using the program NAMD v. 2.9b3.

Contact analysis was performed using home-written routines defining a contact whenever two atoms are closer than 1 Å plus the sum of their van der Waals radii [64].

#### EPAC

The molecular dynamics simulations of the Exchange Protein directly Activated by Cyclic AMP (EPAC), which have been analysed in the present work, have been previously described by Melacini and coworkers [28]. The simulated portion of the protein entails residues 280 to 612 and 643 to 990, i.e. all the catalytic region except for the flexible, unresolved, loop 613–642 connecting the REM and the RA domains. Four EPAC states have been simulated: two equlibrium simulations for the cAMP bound (holo-) active state and apo-inactive state, and the two non-equlibrium apo-active and holo-inactive states. The simulations were run for 60 ns and the analysis was conducted on the last 50 ns. All the details about the simulations can be found in ref. [28].

#### OppA-tripeptide complexes

Molecular dynamics (MD) simulations were run on the free OppA protein (pdb code: 1RKM) and its complexes with tripeptides KLK (1B9J), KIK (1B3G), KDK (1B4Z), KNK (1B5I), KVK (1QKB), KAK (1JET), KFK (1B40), KMK (1B32), KGK (1B3L), KSK (1B51), KQK (1B5J), KKK (2OLB), KEK (1JEU), KTK (1B52), KHK (1B3F), KPK (1B46), KYK (1B58), KRK (1QKA), KCK (1B05), KWK (1JEV).

Crystallization water was removed, and the OppA histidines 55, 117, 142, 161, 405, 440 were protonated in all the systems. The protonation state was calculated using the program H++ (available at URL http://biophysics.cs.vt.edu/index.php) [65] at pH 7 and ionic strength 0.05M. The system, neutralised with NaCl was set up in a cubic box with 0.6 nm buffer around the protein and 0.05M ionic concentration. We used amber99 force field with tip4p water [48]. The cutoff for all the energies is 1.2nm. The system energy is minimized with the steepest descent algorithm as implemented in Gromacs. MD simulations were run for 45 nanoseconds (timestep of 0.002ps) for the free protein, its complexes, and the free peptides. We then sampled for the analysis 2,000 snapshots over the last 20ns.

#### *β*2-microglobulin with citrate coated gold nanoparticles

The *β*2-microglobulin structure was taken from the NMR solution structure (PDB id: 1JNJ). All titratable protein side chains, were assigned their standard protonation state at pH 7.7 with H++ [65] corresponding to the experimental pH. Preliminar rigid-body docking simulations were carried out using Brownian dynamics (BD) techniques with the ProMetCS continuum solvent model [66] implemented in the SDA code (available at URL http://projects.villa-bosch.de/mcmsoft/sda/6.00/), from which the preferred binding site of the protein towards the citrate coated gold nanoparticles (cit-AuNPs) were extracted.

Temperature Replica-Exchange (T-REMD) simulations were started from the most representative and populated complexes resulted from rigid-body BD docking. More in details, 20 ns of unrestrained T-REMD of 32 replicas covering the temperature range between 290 and 320 K [67] were run yielding an aggregated simulation time of 640 ns. All simulations were based on the GolP [68] force field with the SPC/E water model as implemented in the GROMACS package [69]. The lengths of bonds were constrained with the LINCS algorithm. Surface gold atoms and bulk gold atoms were frozen during all simulations but gold dipole charges were left free. Periodic boundary conditions and the Particle-Mesh-Ewald algorithm were used. A 2 fs integration time step was used. Analysis of the trajectories was performed over the last 10 ns of simulations. More details on the method and the model for the cit-AuNPs can be found in Brancolini et al. [70].

## Results

### Conformational entropy of unfolded proteins

In order to compute conformational entropy changes upon folding it is necessary to have a reference for the unfolded state. An explicit simulation of the unfolded state of a polypeptide chain would be unpractical because of both equilibrium constant, dictating the relative population, and kinetic constants coupled with free energy landscape, dictating the length of the simulation time.

**Fig 1:**
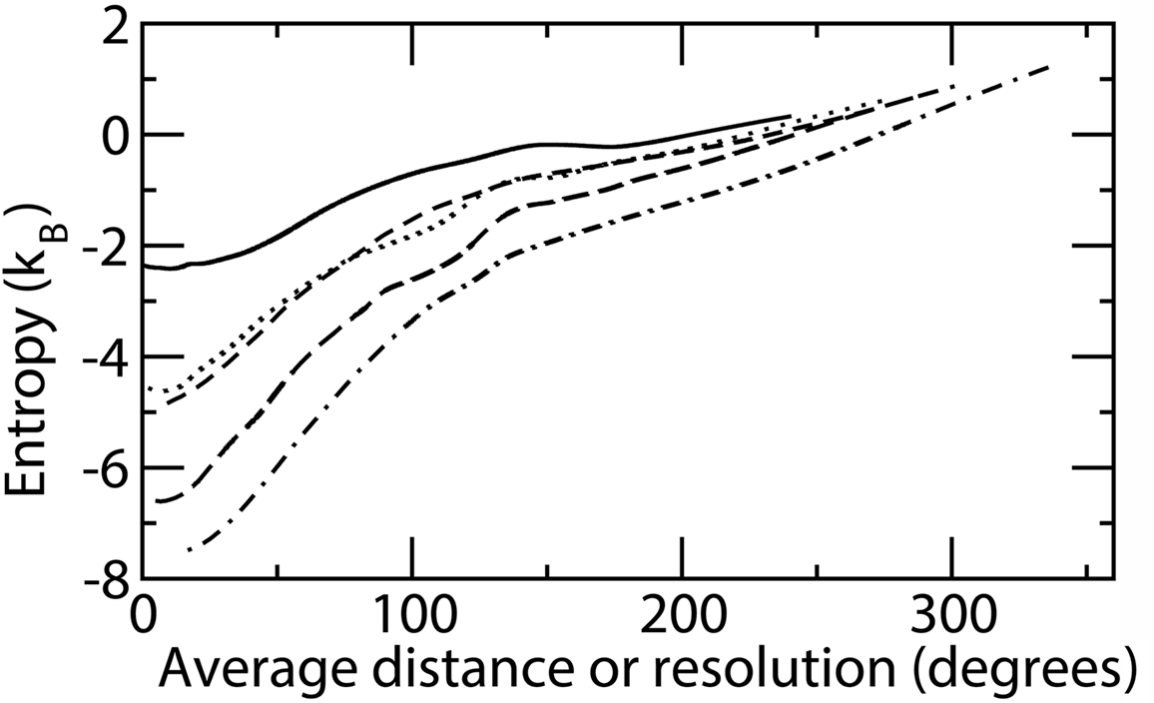
Entropy for residues in unfolded proteins calculated according to the *k* ^*th*^ nearest neighbor vs. the average distance of the *k* ^*th*^ nearest neighbor. Distances are increasing with *k*. The curves are relative to alanine (continuous line), valine (dotted line), isoleucine (short dashed line), tryptophane (long dashed line) and lysine (dot dashed line).

Reference entropy values computed here may be used for computing conformational entropy differences from simulations of folded proteins, assuming, as usual, that aminoacid conformations in the unfolded state are essentially the same for all unfolded proteins. The assumption is in line with the typical lack of any secondary chemical shift observed in NMR spectra of unfolded proteins [71]. Conformational entropies for unfolded proteins (with reference to uniform torsional angle distribution) have been estimated from the conformation of aminoacids in public structural databases, as motivated in the Materials and methods section. Residues from the culled pdb dataset [51] not involved in secondary structures were taken as representative of unfolded conformations. The number of samples for the different aminoacids within protein sequence is reported in Table 1, together with entropies estimated from the nearest neighbor distances. Backbone angles *ϕ* or *ψ* were removed from the set of torsional angles to simulate residues at N- and C-termini, respectively. The entropy computed taking different *k*-nearest neighbors was reasonably consistent with the adopted choice of 1st-nearest neighbors. We report in Fig 1 the computed entropy for the alanine residue (only two torsional angles, *ϕ* and *ψ*, both with 2*π* periodicity) and those of valine, isoleucine, tryptophane and lysine (3, 4, 5 and 6 torsional angles, respectively) versus the average distance of the nearest-neighbors which increases as *k* increases. The entropy versus distance is reported instead of the entropy versus *k* because the average distance of the *k*-nearest-neighbors represents the resolution of the corresponding histogram approach to probability and entropy computation. As expected with increasing average distances details of the distribution are lost and the entropy increases. As the average distance approaches half of the minimum periodicity of the torsional angles, corrections should be made to the reference hyperspherical volume implied in Eq (9). In the absence of corrections artifactual curve behaviour is apparent. In the figure the onset of such effects takes place at 180 degrees. The entropy computed by the present method cannot be compared straightforwardly with the many estimates of sidechain entropy reported in the literature [72] because here the distribution of all residue torsional angles are considered together. In an approximate analysis we subtracted the entropy corresponding to alanine (as a model for all aminoacids backbone) from all other entropies and compared the resulting values with the values calculated by Koehl and Delarue [73]. The results correlate well (r = 0.86), although the estimated entropy is on average 1.8 times larger, most likely because of finer resolution due to larger database and different method. The trend of computed entropy with average nearest neighbor distance is observed in Fig 1.

**Table 1:** Entropy and average nearest neighbor distance of unfolded aminoacids in *k*_*B*_ units. The row CYS (*ϕ*, *ψ*, *χ*_1_) refers to cysteines involved in disulfide bridges. For N- and C-terminal residues see text.

			N-terminal		C-terminal		
aa	Entropy	av. dist.	Entropy	av. dist.	Entropy	av. dist.	counts
ALA	−2.4	0.5	−0.6	0.0	−1.0	0.0	23859
CYS	−4.7	15.0	−3.1	9.2	−3.3	8.5	845
CYS (*ϕ*, *ψ*, *χ*_1_)	−3.6	4.7	−2.1	1.5	−2.3	1.3	3250
ASP	−4.5	5.5	−2.7	2.6	−2.9	2.4	27673
GLU	−5.2	11.3	−3.5	7.4	−4.0	6.7	19087
PHE	−4.9	7.0	−3.2	3.6	−3.7	3.1	11603
GLY	−1.9	0.5	−0.4	0.0	−0.5	0.0	43795
HIS	−4.4	8.5	−2.7	4.6	−3.1	4.0	8991
ILE	−6.6	4.8	−4.9	2.3	−4.9	2.2	12908
LYS	−7.5	16.8	−6.0	12.5	−6.3	11.6	18930
LEU	−6.3	4.5	−4.6	2.1	−5.2	1.8	22763
MET	−6.1	14.7	−4.5	10.1	−4.9	9.1	4848
ASN	−4.7	6.6	−3.0	3.3	−3.1	3.1	20987
PRO	−0.8	0.0	−0.8	0.0	0.0	0.0	27620
GLN	−5.5	13.7	−3.9	9.3	−4.4	8.3	11567
ARG	−6.9	18.2	−5.3	13.6	−5.7	12.7	15776
SER	−4.6	8.0	−3.0	4.2	−3.2	3.9	10053
THR	−5.1	7.2	−3.4	3.7	−3.6	3.5	8829
VAL	−4.6	2.2	−3.0	0.5	−3.0	0.5	17243
TRP	−4.8	9.2	−3.1	5.1	−4.1	23.5	4332
TYR	−5.4	17.2	−3.9	12.0	−4.2	10.9	3550

### Conformational entropy of a folded protein: *β*2-microglobulin

The conformational entropy change upon folding is obtained in this section as the difference in the conformational entropy computed from molecular dynamics simulation between the folded state of the protein and the reference entropies for the unfolded state computed from database samples in the previous section.

The system chosen here is *β*2-microglobulin which is a small, well characterized protein.

300 snapshots from 30 ns molecular dynamics simulations of *β*2-microglobulin have been converted in torsional angles and entropy was computed, residue by residue. It should be noted that in the approach only correlations among degrees of freedom of the same residues are considered, whereas those among torsional angles of different residues would lead to very high dimensional space. This issue will be addressed in this subsection.

The entropies computed for the model residues in the unfolded protein (see previous subsection) have been subtracted from the computed entropies. The plot of computed entropy vs. residue number is sensitive to the nature of each residue, however, it is worth noting that the first and last residue display small but positive differential entropy. It is likely that this is due to the fact that these are really terminal residues, with more conformational freedom than the model ones, which were taken from residues in the sequence with the terminal torsional angles ignored. For all other residues the individual entropies range from -6.1 *k*_*B*_ for R81 to -0.4 and -0.7 *k*_*B*_ for R89 and K15 in the mobile FG and AB loop, respectively. It is reassuring that most mobile residues show entropies close to those of the unfolded residues.

The residues displaying the largest differential entropy are mostly located in stable regions of the protein. For instance, R81 (-6.1 *k*_*B*_) is close to C80 involved in a disulfide bridge in the most stable region of the protein. R81 is partly exposed to the solvent but is involved in a salt bridge with residue D38. The picture is however somehow obscured by the individual nature of residues. In order to overcome this difficulty we consider the sum of entropies of residues clustering together. To this end we list for each residue the contacting residues and sum their computed entropies as representative of the entropy corresponding to a local opening reaction involving that residue. By local opening we mean the process of partial or global unfolding, where an amide hydrogen becomes exposed to the solvent, whose global entropy is measured e.g. by the Bluu-Tramp hydrogen-exchange NMR experiment [3, 4].

**Fig 2:**
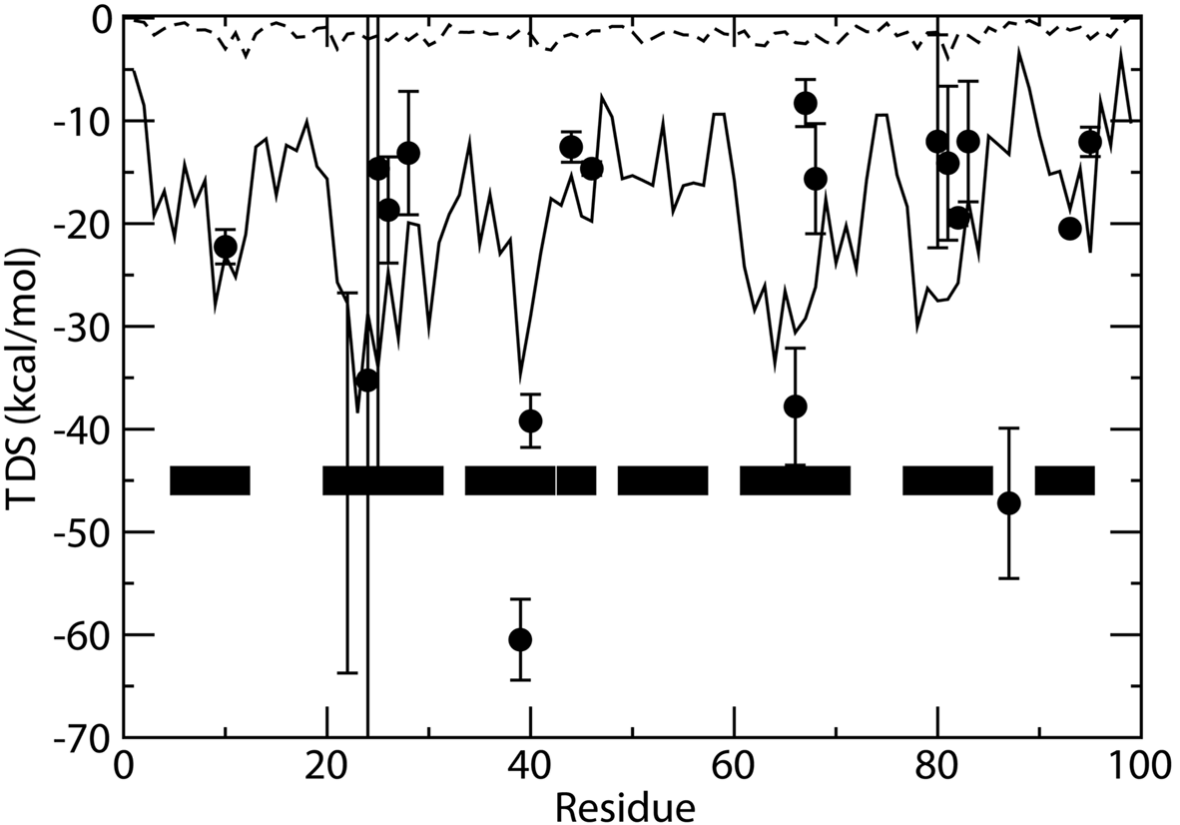
Entropy versus residue number from 30 ns *β*2-microglobulin molecular dynamics simulation. Residue computed entropy (dashed line), summed entropy corresponding to residue neighborhood (continuous line). In the low part of the figure residues with defined secondary structure are indicated by black boxes. Black dots with error bars represent experimentally determined entropy from Bluu-Tramp hydrogen-deuterium exchange NMR experiments [3]. The latter experiments measure the enthalpy and entropy of the process (partial or global unfolding) which exposes protein amide hydrogens to solvent.

The results are reported in Fig 2 where the single residue and the cluster (i.e. the sum over neighboring residues) entropies are reported, together with experimental results. The latter include not only the conformational but also solvation entropy, and serve only the purpose to show the scale of the phenomena under consideration. It is interesting to note that the experimental entropy values which are most largely different from the conformational ones are those for the only three leucines for which experimental information is available, L39, L40 and L87 and the two aromatic residues F22 and Y66. For these residues hydrophobic solvation contributions to entropy are expected to be relevant. For other hydrophobic or aromatic residues data (Y10, Y26, Y67, V82, V93, I46, W95) the same large difference is however not observed, pointing out the importance of specific details of local opening rather than a general hydrophobic effect, which are not captured by our simple model for local openings.

It should be noted that the approach followed here neglects correlations between degrees of freedom belonging to different amino acids. For a complete treatment of such correlations, approaches have been developed by Gilson and coworkers [15, 74]. The correlation within the same amino acid are expected to be large and actually many studies of protein conformations have demonstrated the correlation between secondary structure and side chain torsional angles [75]. It is however true that backbone angles of adjacent residues show often correlated motions which preserve the overall direction of the chain [76]. Such correlations have been observed very early in molecular dynamics simulations [77]. In order to assess the magnitude of this effect we computed the entropies *S*_*ϕ*_*i*__ and *S*_*ψ*_*i*−1__ corresponding to the 1-dimensional distribution of angles *ϕ*_*i*_ and *ψ*_*i*−1_ separated by the peptide bond and the entropy *S*_*ϕ*_*i*_, *ψ*_*i*−1__ corresponding to the 2-dimensional joint distribution. The difference (*S*_*ϕ*_*i*__+*S*_*ψ*_*i*−1__−*S*_*ϕ*_*i*_, *ψ*_*i*−1__), i.e. the mutual information of *ϕ*_*i*_ and *ψ*_*i*−1_ [74] was found to be positive, as expected, and on average of small entity (0.13 ± 0.05 *k*_*B*_). An extreme test for contacting side chains is provided by the degrees of freedom of cysteines involved in a disulfide bridge, where a strong correlation is expected. Also here the explicit calculation of the entropy corresponding to the two cysteines compared to the two isolated residues gave a positive difference (1.5 *k*_*B*_) which however amounts to ca. 10% of the overall entropy of the two residues, before subtraction of free residues one, and 30% of the entropy after subtraction. We expect that in the absence of restraints on chain direction in the unfolded state, the correlation should be rather small. A similar test was performed for all torsional angles of R81 with all other torsional angles of *β*_2_-microglobulin. The average mutual information is about 0.06 *k*_*B*_. Overall these data suggest that correlations, other than those within the same aminoacids may give corrections to the conformational entropies which could be in the range of one order of magnitude smaller than the single amino acids conformational entropies of folding.

**Fig 3:**
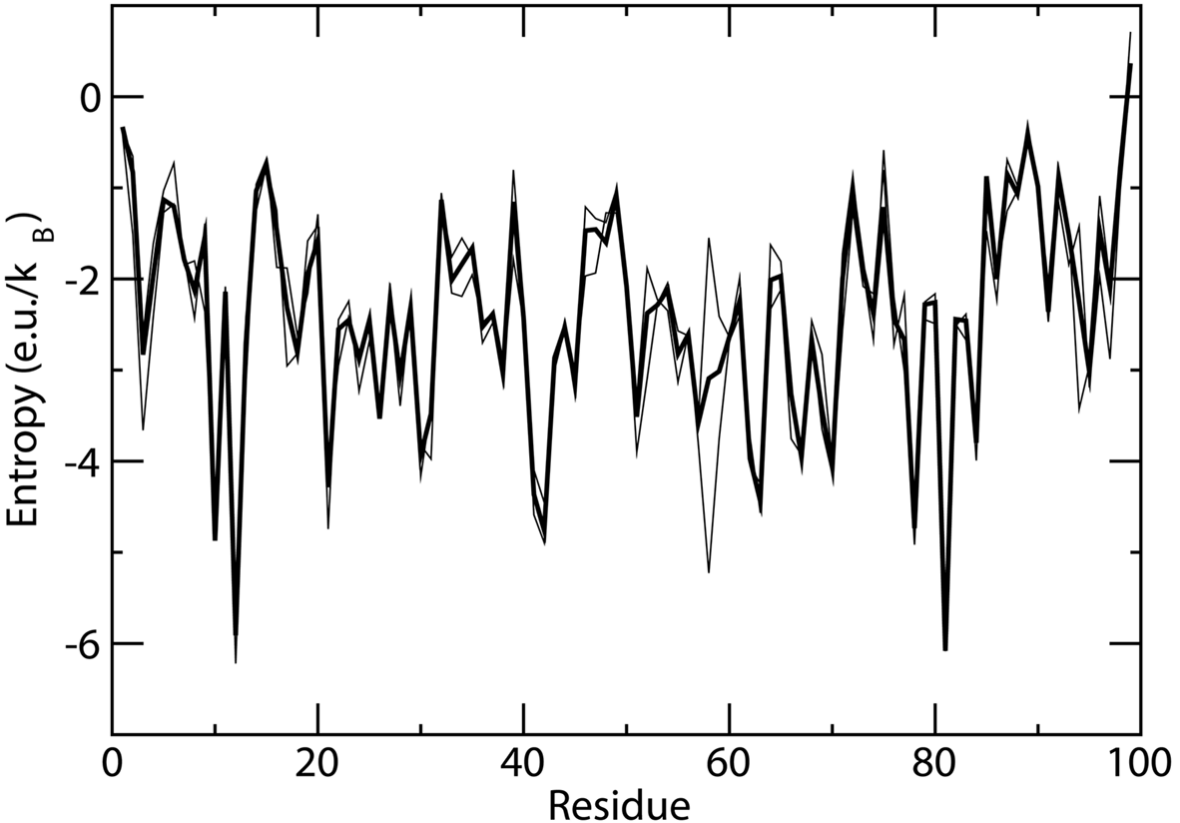
Entropy versus residue number from 30 ns *β*2-microglobulin molecular dynamics simulation (thick line) and from the first and second half of the simulation (thin lines).

For this rather short simulation we checked also convergence. The trajectory was divided in two and the results for the separated halves were plotted together with the results for the total simulation time. As it can be seen in Fig 3 the results are rather stable with few exceptions. The largest difference is observed for K58 which loses close interaction with D59 in the second half of the simulation becoming more flexible.

### Conformational entropy and order parameters

In this section we address the relationship between conformational entropies and order parameters, which are measured by NMR typically on HN vectors and are taken as a measure of conformational dynamics of each aminoacid.

The system chosen for this purpose is the exchange protein directly activated by cAMP (EPAC) which is a receptor undergoing functionally critical conformational and dynamics changes upon binding cAMP.

Changes in dynamics have been monitored by changes in HN order parameters, as measured by NMR, used, in turn, to measure changes in conformational entropy.

Four simulations, starting from the active and inactive conformations in the apo and holo forms, were previously performed, analysed and compared with experimental data by Melacini and coworkers [28]. We use the distance-based estimates of entropies for the four trajectories, which compared well with experimental data, and use this example to address the following issues:

1. how well the order parameters *S* ^2^, typically measured on HN vectors using NMR, correlate with the entropy associated with backbone degrees of freedom;
2. how well the the entropy associated with backbone degrees of freedom correlates with the entropy of all torsional degrees of freedom.

**Fig 4:**
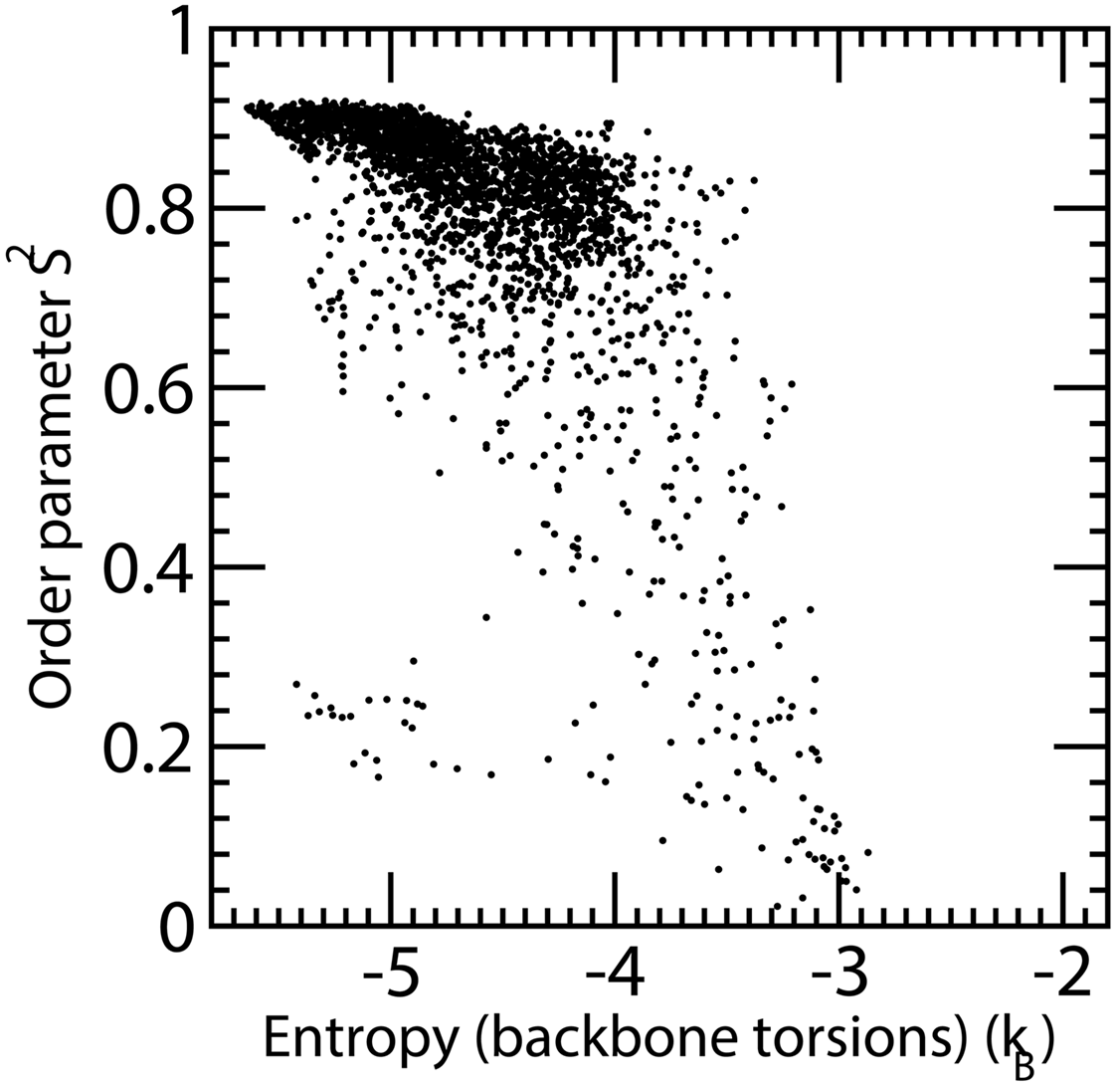
HN vector order parameter versus the computed backbone entropy on all four EPAC MD trajectories. The single simlations show some differences, but deviations from a sigmoidal behaviour is found for all four simulations.

For the first question, a straightforward correlation of the order parameters of the HN vector with the conformational freedom of backbone torsion angles is not expected because the HN vector could be kept in place e.g. by a hydrogen bond even with floppy flanking peptide moieties. Rather, an agreement between order parameters and entropies averaged over chain segments should be expected. Indeed the raw correlation between backbone entropy and the order parameter computed on MD trajectories is poor, besides a necessary transformation between unbounded entropy and bounded order parameter. However, when the backbone entropy is smoothed using the lowess algorithm [78] implemented in the software R [79], with the parameter f set to 0.02, the (anti)correlation increases to -0.61 (Fig 4). The plot of order parameter versus the computed backbone entropy shows a semiquantitative relationship between the two. Notwithstanding the very different assumptions underlying the present calculation and the harmonic oscillation model used by Wand and coworkers [80], the points are scattered about a sigmoidal curve, thus confirming the insight provided by the original link between order parameters and associated entropy.

The changes in the order parameter *S* ^2^ occurring upon conformational transitions may be associated, based on the concept that they reflect overall conformational freedom changes, to changes in conformational entropy. For this reason we examine how backbone entropy, which is semiquantitatively measured by the order parameter *S* ^2^, is correlated with overall conformational entropy. In other words we wish to estimate the latter from the measured HN order parameter *S* ^2^.

**Fig 5:**
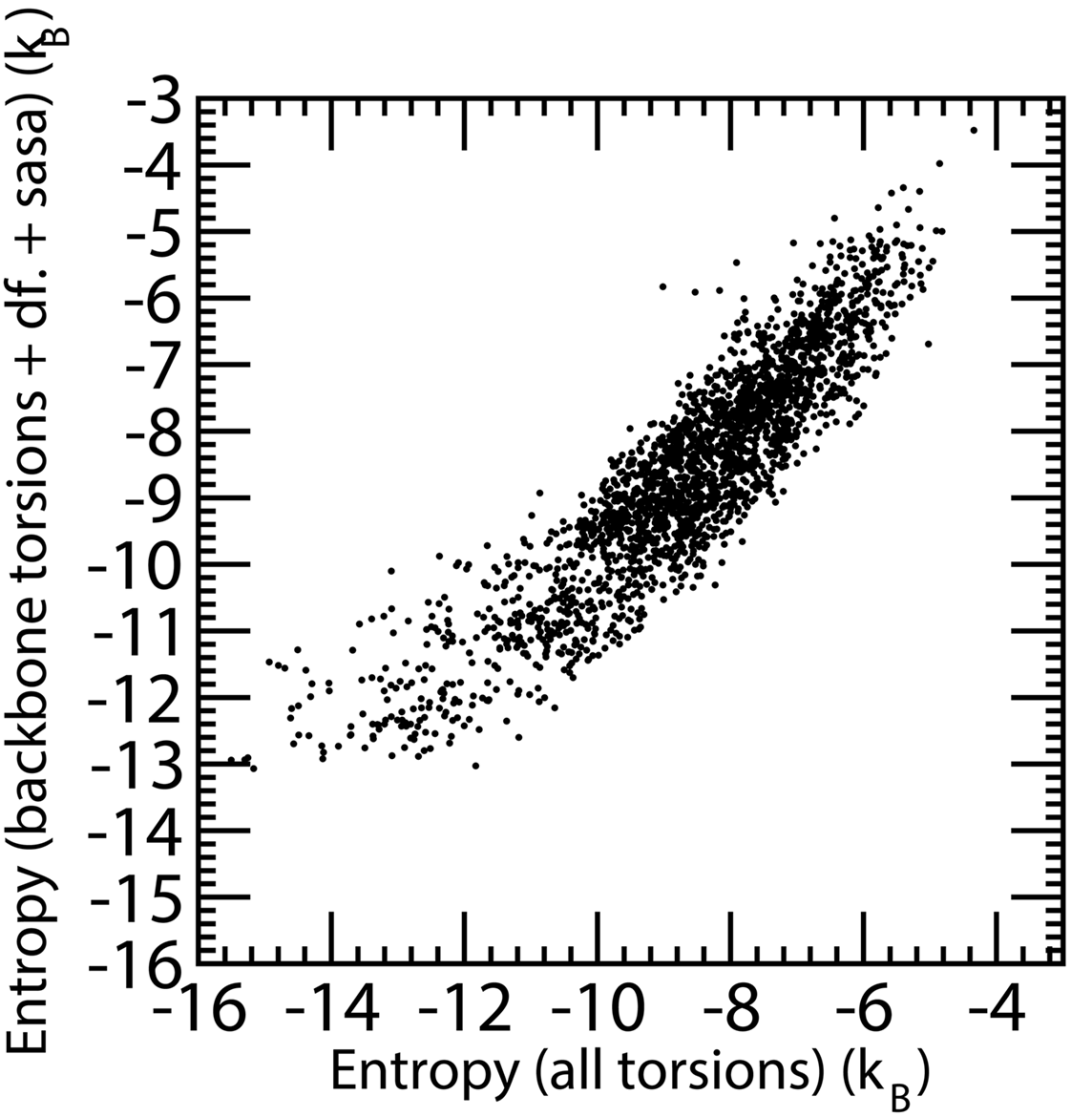
Residue entropy computed for all four EPAC MD simulations. On the x-axis the entropy is computed using all residue torsion angles, on the y-axis the entropy is fitted as a linear combination of backbone entropy, the number of sidechain degrees of freedom and the solvent accessible surface area of the residue. Glycine, Alanine and Proline residues that do not possess sidechain torsion angles, are not reported in the figure.

We computed the entropy associated with backbone and all torsional angles for all four simulations and fitted the overall conformational entropy as a linear combination of backbone conformational entropy (coefficient 1.120) and the number of sidechain rotational degrees of freedom (coefficient -1.425), assuming implicitly that each degree of freedom gives a similar entropic contribution. Alternatively the difference between the absolute entropy of all degrees of freedom and backbone degrees of freedom could be fit as linearly dependent on the number of sidechain degrees of freedom (coefficient -1.633) with similar results. Residues alanine, glycine and proline that do not possess torsions other than backbone ones were removed from the comparison. The fit shows a strong correlation with fitted data (correlation coefficient 0.859). This analysis does not take into account whether the residue is exposed or not. A slight modification of the approach considers also the exposed area of the residue. In this case the estimated absolute entropy of the residue is a weighted sum of the backbone entropy (weight: 1.08), of the number of degrees of freedom of the sidechain (weight: -1.78) and the exposed surface area in Å^2^ (weight: 0.012). With this simple linear model the correlation coefficient increases to 0.906 and the root mean square deviation of the predicted versus computed absolute entropy is 0.76 *k*_*B*_ (Fig 5).

### Rototranslational entropy loss upon binding: OppA-tripeptide complexes and *β*2-microglobulin association with a citrate coated gold surface

The free energy of binding of tripeptides with the E. coli peptide protein binding has been extensively studied by computational methods [30]. We address here the rototranslational entropy loss upon binding. We first select an almost rigid three-atom fragment which is most decoupled from internal degrees of freedom of the peptide. To this end we consider the N-CA-C fragment of each of the three aminoacids and we compute the root mean square fluctuations of the three atoms upon superposition of the OppA regions involved in binding. All three aminoacids gave similar results, depending on the specific tripeptide, and therefore we kept the central aminoacid backbone atoms as the reference triad atoms.

**Fig 6:**
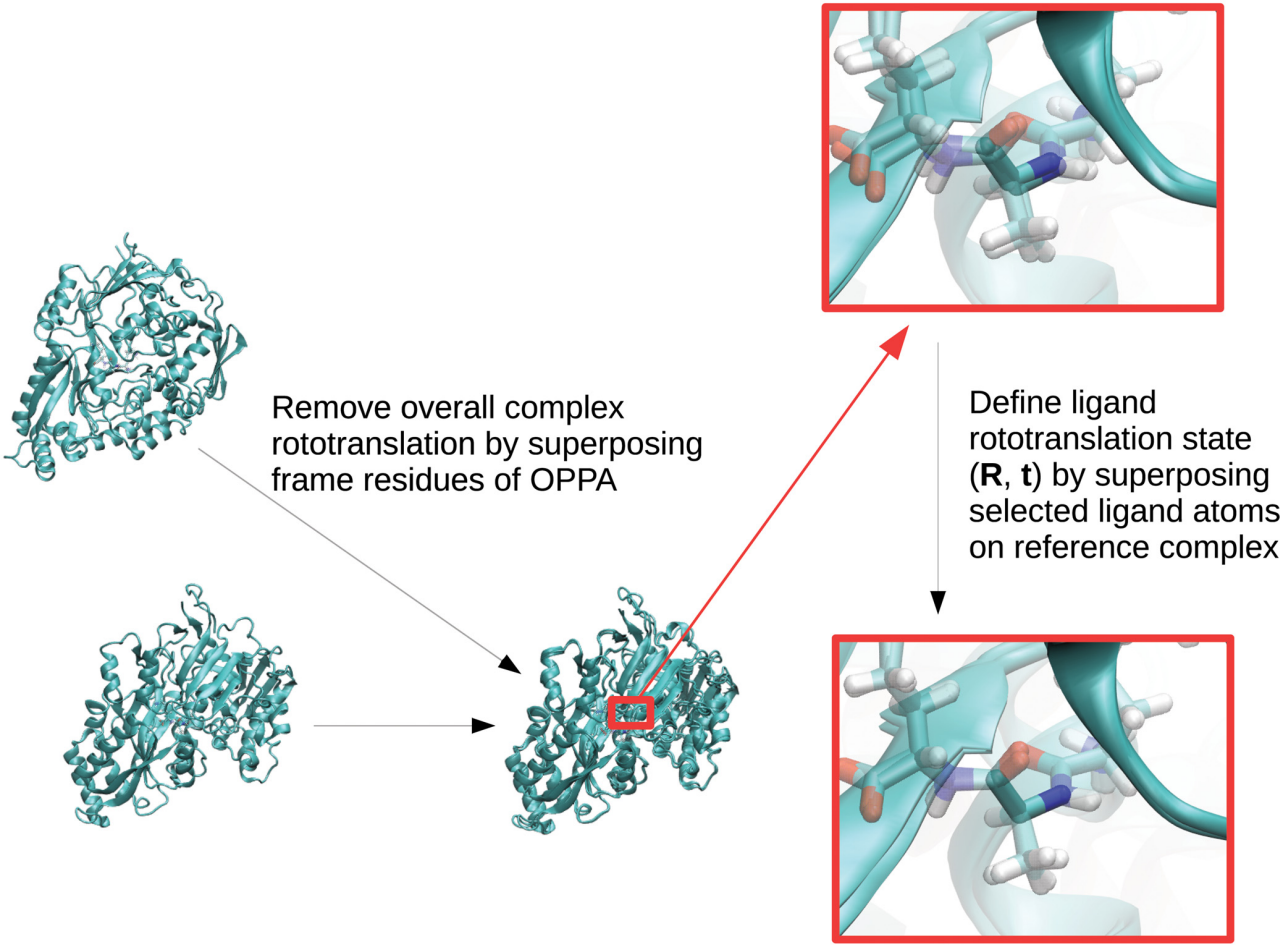
Graphical representation of how the rotation matrix and translation vector describing the rototranslational state of the tripeptide ligand with respect to OppA is obtained. First, overall rototranslation of the complex is removed by superposition of frame residues. Second, the central residue N, CA and C atoms are superimposed on the reference structure. The resulting rotation matrix and translation vector describe the rototranslational state of the tripeptide ligand.

Overall complex rototranslation and the movements internal to OppA protein were removed by superimposing backbone atoms of residues 32–37, 245–246, 269–271, 397–404, 414–419 of the OppA protein to the starting structure. The superimposed structure was then used for superposition of the three reference ligand backbone atoms and the resulting rotation matrix and translation vector were written. This procedure is illustrated in Fig 6.

The resulting computed entropies are reported in Table 2. The reference states are the randomly oriented rigid triad of backbone atoms (*S* = −*k*_*B*_log(4*π* ^2^)), for the rotational state, and the 1M concentration state, for the translational state (−*k*_*B*_log(1/1660)), with angles measured in radians and distances in Å. The resulting rototranslational entropy of association at room temperature is ca. 10 kcal/mol. A similar value was predicted earlier based on semiquantitive considerations by others (e.g. [39, 81]).

**Table 2:** Rotational and translational entropies in *k*_*B*_ units for the binding of 20 tripeptides to OppA protein. In parentheses values computed using the histogram method are reported. The average nearest-neighbor distance (in Å for translations and in rad for rotational distances) is reported in Av. dist. columns.

peptide	Rot. entropy	Av. dist.	Transl. entropy	av. dist.
KAK	−8.17 (-8.44)	0.017	−8.81 (-8.84)	0.04
KCK	−8.73 (-8.74)	0.014	−9.03 (-9.00)	0.04
KDK	−8.34 (-8.45)	0.016	−9.11 (-9.14)	0.03
KEK	−8.76 (-8.73)	0.014	−9.17 (-9.17)	0.03
KFK	−8.83 (-8.90)	0.014	−9.10 (-9.17)	0.03
KGK	−8.09 (-8.33)	0.018	−8.40 (-8.56)	0.04
KHK	−8.85 (-8.94)	0.013	−9.31 (-9.29)	0.03
KIK	−7.62 (-8.01)	0.020	−7.98 (-8.22)	0.05
KKK	−8.99 (-8.98)	0.013	−9.36 (-9.26)	0.03
KLK	−8.04 (-8.08)	0.018	−7.92 (-8.16)	0.05
KMK	−8.68 (-8.77)	0.014	−9.14 (-9.15)	0.03
KNK	−8.40 (-8.48)	0.016	−9.04 (-8.98)	0.04
KPK	−8.55 (-8.64)	0.015	−9.28 (-9.26)	0.03
KQK	−8.68 (-8.78)	0.015	−9.12 (-9.11)	0.03
KRK	−9.00 (-8.89)	0.013	−9.64 (-9.48)	0.03
KSK	−8.30 (-8.53)	0.016	−8.86 (-8.84)	0.04
KTK	−8.63 (-8.82)	0.015	−8.98 (-8.98)	0.04
KVK	−8.86 (-8.90)	0.014	−9.33 (-9.16)	0.03
KWK	−8.90 (-8.92)	0.013	−9.27 (-9.17)	0.03
KYK	−8.71 (-8.76)	0.014	−8.90 (-8.85)	0.04

Rotational and translational entropies have been computed using also the histogram method for the same set of 1000 snapshots. The results are dependent on the number of bins used. The latter was chosen as to limit the number of bins containing a single sample. With this choice the agreement is remarkable with correlation coefficients over 0.96 and mean square root difference of less than 0.2 *k*_*B*_ (Table 2).

In order to study a less tightly bound system we consider *β*2-microglobulin non-covalently bound to a gold nanoparticle coated with citrate. Here the protein is limited in its movements along the axis perpendicular to the surface plane and in its orientations. We have considered the distance of the center of geometry of the most structured regions from the plane through the oxygen atoms of the citrate bound to the gold surface. The reference rotation state is computed through superposition of the backbone atoms of the residues that contact most the surface, i.e. I1, K3, K58, W60 with the corresponding atoms in the first structure. After setting a common reference frame for the bound state, the rotation matrix is found by superposition of secondary structure elements. Because the bound state is defined irrespective of the orientation and position over the plane, the computed rotational entropy must be computed with a modified formula taking into account the reduction in dimensionality. The translational entropy is computed by Eq (9) with dimension one and taking the distance between two sample points as the difference in the distance from the surface plane.

Similarly for the rotational entropy of the molecule in contact with the surface it should be considered that it should be corrected by an additional term *k*_*B*_log(2*π*) to take into account any rotation about the axis perpendicular to the surface plane. With these corrections the overall translational and rotational entropy change from the 1M reference state to the surface bound molecule amounts to -2.1 *k*_*B*_ and -4.8 *k*_*B*_, respectively. These figures, which are considerably smaller than the corresponding ones for tight binding, could be underestimated because of the expected correlation between translational and rotational degrees of freedom for the bound state.

## Discussion

In this work we have applied a recently developed method to estimate entropies based on the distances between conformational samples of proteins. We have considered a set of systems representative of important processes like protein folding and binding, where the computation of entropies may be problematic, e.g. when the distribution of conformational variables is multimodal and/or when the dimensionality and the number of samples prevents the use of histograms.

We have first derived reference entropies for single aminoacids in unfolded proteins. Conformational samples have been taken from the regions not involved in secondary structure elements in a non-redundant database of 3600 protein structures.

The set of reference entropies has been subtracted from the entropies computed from molecular dynamics simulation snapshots to obtain folding entropy contributions from each aminoacid of *β*2-microglobulin. The latter entropies (locally summed) have been compared with NMR measured entropies (which entail also solvation entropy) showing that the conformational entropy gives an important contribution to the measured data.

The effect of correlations between residues appears limited, at least as judged from the few examples reported in the Results section.

*S* ^2^ order parameters, which are often taken as a probe for local conformational entropy, have been compared with the entropy estimated for four molecular dynamics simulations of different EPAC states showing that:

- HN order parameter is correlated with the entropy of backbone degrees of freedom;
- the relationship has the sigmoidal trend hypothesized by Wand and coworkers [80];
- the entropy associated with backbone degrees of freedom is well correlated with the entropy of all degrees of freedom, when the number of sidechain degrees of freedom and solvent exposure is taken into account. These results support therefore the use of HN vector order parameter as a probe for overall entropy.

The rotational and translational entropy loss upon protein-ligand binding and upon protein-surface binding have been estimated, resulting in values compatible with those based on simpler models.

With all the limitations that can be envisaged, including lack of consideration of bond length and angle fluctuations, of correlations among degrees of freedom, poor sampling of conformational space, the estimate of entropy based on nearest neighbor distance provides in all the presented examples insight into the conformational and configurational entropy of the processes considered. The method can be readily applied to molecular dynamics snapshots and provides an entirely alternative route to estimate entropy where other more traditional methods based on histograms or on variance-covariance matrix of fluctuations may prove difficult to apply or less accurate.
